# Immune Response to “Self” Lens in *Xenopus laevis* Enucleated during Larval Life

**DOI:** 10.1155/1999/19741

**Published:** 1999

**Authors:** Takeshi Enomoto, Shin Tochinai

**Affiliations:** 1 Division of Biological Sciences Graduate School of Science Hokkaido University Sapporo 060-0810 Japan

**Keywords:** *Xenopus*, development, immune reaction, lens, self

## Abstract

We have reinvestigated an important issue in the amphibian immunology that has not been settled for years since the pioneer work of Triplett, concerning the necessity of being exposed to organ-specific antigens early in development. It was found that syngeneic lenses were rejected by frogs, *Xenopus laevis*, that had been enucleated (eye removed) during early larval life. This rejection did not occur in intact frogs or in those enucleated in later larval or adult life. Whereas the splenocytes from intact frogs did not proliferate in response to a co-cultured syngeneic lens, those from frogs that had been enucleated at any of the larval stages, or even after metamorphosis, proliferated intensely. Both of these responses were shown to be thymus- dependent. In conclusion, it was demonstrated that the frog immune system rejected even syngeheic lenses by enucleation in early larval life and that it began to recognize the syngeneic lenses by lymphoid proliferation after enucleation, even in later life.


*Developmental Immunology*, 1999, Vol. 7(1), pp. 23-32
Reprints available directly from the publisher
Photocopying permitted by license only

© 1999 OPA (Overseas Publishers Association)
N.V. Published by license under the
Harwood Academic Publishers imprint,
part of Gordon and Breach Publishing Group
Printed in Malaysia

### Immune Response to "Self" Lens in *Xenopus laevis* Enucleated during Larval Life

TAKESHI ENOMOTO and SHIN TOCHINAI*

*Division of Biological Sciences, Graduate School of Science, Hokkaido University, Sapporo 060–0810, Japan*

*(Received 27 February, 1998; Revised 14 July, 1998)*

We have reinvestigated an important issue in the amphibian immunology that has not been settled for years since the pioneer work of Triplett, concerning the necessity of being exposed to organ-specific antigens early in development. It was found that syngeneic lenses were rejected by frogs, *Xenopus laevis*, that had been enucleated (eye removed) during early larval life. This rejection did not occur in intact frogs or in those enucleated in later larval or adult life. Whereas the splenocytes from intact frogs did not proliferate in response to a co-cultured syngeneic lens, those from frogs that had been enucleated at any of the larval stages, or even after metamorphosis, proliferated intensely. Both of these responses were shown to be thymus-dependent. In conclusion, it was demonstrated that the frog immune system rejected even syngeheic lenses by enucleation in early larval life and that it began to recognize the syngeneic lenses by lymphoid proliferation after enucleation, even in later life.

*Keywords:* *Xenopus*, development, immune reaction, lens, self

### INTRODUCTION

It is generally accepted that the immune system is inherently incapable of discriminating foreign and self antigens but can "learn" during development not to respond only to self antigens. If this theory is correct, self antigens that are removed prior to development of the immune system cannot be regarded as "self". The first experimental support of this was reported as early as 1962 by Triplett (1962). In his experiment, complete removal of the embryonic hypophysis was followed by rejection of the reimplanted "self" hypophysis in larval tree frogs (*Hyla regilla*). On the other hand, the results obtained in another amphibian were in marked contrast to those by Triplett. From *Xenopus laevis* embryos, the hypophysis, the thyroid, or the eye anlagen were removed, but the animals never rejected syngeneic implants of removed organs from the same strain in later life (Rollins-Smith and Cohen, 1982a, 1982b, 1983, 1990; Rollins-Smith et al., 1990; Maeno and Katagiri, 1984).

This situation prompted us to reexamine Triplett's experiments, by utilizing a syngeneic strain of a frog *(Xenopus laevis).* We selected a lens for a target tissue for two reasons: (1) It is technically feasible to remove all of the tissues expressing the antigens without disturbing larval development and later growth,

* Corresponding Author. Tel.: +81-11-706-5293. Fax: +81-11-757-5994.

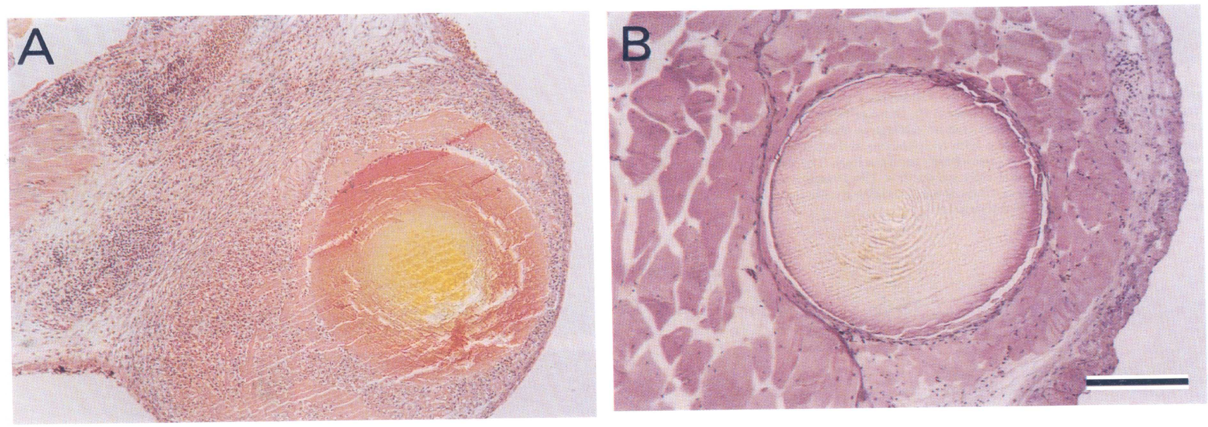

FIGURE 1 Inflammatory tissues formed around the grafted lens 20 days after grafting. Intense inflammation with lymphocytes and phagocytes occurred only around the lenses that were grafted in frogs enucleated at stage 43 (A) but not in frogs enucleated at the same stage and thymectomized soon after at stages 45 to 47 (B). (Bar, 200 micrometer). (see colour plate I at the back of this issue)

and (2) it can be removed at any time even before the immune system commences to develop. Thus, the mode of immune response to a "self" lens in metamorphosed frogs that had been enucleated (eye removed) during ontogeny was investigated. We found that the syngeneic lenses were rejected only by frogs that had been enucleated during early larval life. In contrast, the splenic lymphocytes from frogs enucleated at any larval stage, or even after metamorphosis, proliferated intensely in response to the co-cultured syngeneic lens after its removal.

### RESULTS

### Eye or Lens Transplantation

To determine when self-tolerance is naturally induced to lens antigens during ontogeny, we transplanted syngeneic eyeballs or lenses in frogs that had been enucleated at different larval and adult stages, and observed the immune responses against the grafted lenses. At first, whole syngeneic eyeballs were transplanted in 5 intact adult frogs as well as those that had been enucleated at stage 43 (6 frogs), 56 (5 frogs), or 66 (6 frogs). However, no apparent macroscopic and histological damage in the lenses or the other tissues in the eyeballs was observed by the 20$^{th}$ day after transplantation.

Since it is highly plausible that the eye is an immunologically privileged site in *Xenopus* as in mammals, we modified the experimental protocol by transplanting only an isolated lens rather than a whole eyeball. After this modification, we found that in lenses transplanted in adult frogs that had been enucleated at stage 43, the lens epithelial cells suffered destruction and eventually disappeared (Figs. 1 and 2). On the 20$^{th}$ day after transplantation, destruction with surrounding inflammatory cell accumulation was observed histologically in 3 out of 5 transplanted lenses, and in the other 2 lenses, epithelial cells had already disappeared by this time. On the 50$^{th}$ day after grafting, there were no longer any residual epithelial cells in all 4 lenses observed (Table I). Although phagocytes were observed to take up pieces of lens fiber frequently, a major mass of lens fiber remained even on the 100$^{th}$ day after grafting (data not shown).

No destruction or disappearance of lens epithelial cells was observed histologically in intact adult frogs, in frogs with only one eye removed at stage 43, or in frogs that were later enucleated bilaterally, except for two rare cases: one in a frog enucleated unilaterally at stage 43 and another in a frog enucleated bilaterally at stage 56 (Table I and Fig. 2B). In frogs enucleated at

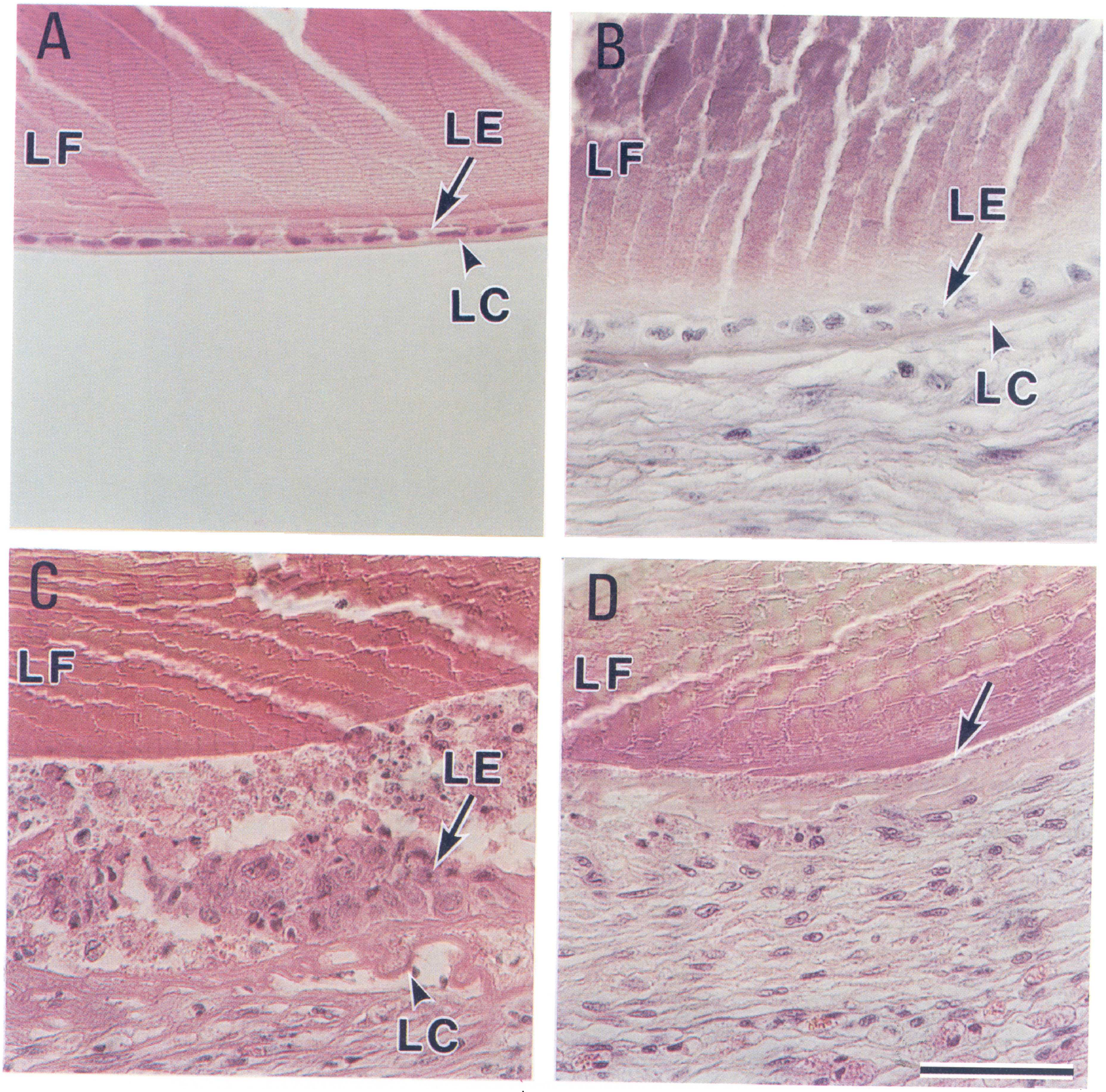

FIGURE 2 Histological status of the transplanted lenses. (A) Intact lens before transplantation consists of the lens epithelium (LE: arrow), lens capsule (LC: arrowhead), and lens fiber (LF). Damage to the transplanted lens was defined histologically as follows. (B) No damage: The transplanted lens is embraced by fibroblasts, but no damage is observed histologically in the lens. Few leukocytes are accumulated. (C) Destruction of the lens epithelial cells: Inflammation with lymphocytes and other leukocytes is observed around the transplanted lenses. Many leukocytes migrate between the up lifted lens capsule (arrowhead) and lens fiber. The lens epithelial cell layer detached from the lens fiber is fragmented and stratified. The nuclei of the lens epithelial cells are swollen. (D) Disappearance of lens epithelial cells: The lens epithelial cells have disappeared completely. The arrow represents the place where the lens epithelial cells should exist in a normal lens. In some cases, inflammatory cell accumulation is still observable at this stage. (Bar, 50 micrometer). (see colour plate II at the back of this issue)

stage 43 and thymectomized at stages 45 to 47, destruction with inflammation and disappearance of the lens epithelium did not occur except for one case observed on the $50^{th}$ day (Table I). In this case, the damage was not very severe, and the major part of the cells remained almost intact.

TABLE I Destruction and Disappearance of lens epithelial cells

| *Frogs* | *20 days* | | | *50 days* | | |
|---|---|---|---|---|---|---|
| | *Destruction* | *Disappearance* | *Total* | *Destruction* | *Disappearance* | *Total* |
| Enucleated at stage 43 | 3 | 2 | 5 | - | 4 | 4 |
| Unilaterally enucleated at stage 43* | 0 | 0 | 6 | 1 | 0 | 7 |
| Enucleated at stage 43 and thymectomized at stage 45–47 | 0 | 0 | 4 | 1 | 0 | 5 |
| Enucleated at stage 56 | 1 | 0 | 4 | 0 | 0 | 5 |
| Enucleated at stage 66 | 0 | 0 | 5 | 0 | 0 | 3 |
| Enucleated after metamorphosis | 0 | 0 | 4 | 0 | 0 | 7 |
| Intact* | 0 | 0 | 5 | 0 | 0 | 3 |

#The histopathologic sections of the grafted syngeneic lenses were observed on the 20th and 50th days after grafting. Each number represents the number of grafts in which lens epithelial cells were being destroyed but still remained, or had disappeared among totals.
*Except for these two groups, all animals were enucleated bilaterally.

To specify the cells that had accumulated around the grafts, the transplanted lenses with surrounding tissues were stained immunohistochemically using T-cell-specific mAbs XT-1 or CD8-specific mAbs AM22 on the 20th day after grafting. It was found that massive T lymphocytes had accumulated and that many of them were $CD8^+$ in the inflammatory tissues.

### Proliferative Response of Splenocytes

Next, we co-cultured a lens and splenocytes to assess the magnitude of the responses of the immune cells against the lens antigens *in vitro*. The proliferative response of splenocytes against the lens of the semi-xenogeneic frogs made as a preliminary experiment showed its highest value in the 4th to 7th day in co-culture (data not shown). Thus, BrdU incorporation into splenocytes that were cultured with the syngeneic lens were examined during 24 hours from the 4th day. Although splenocytes from intact adult frogs displayed positive proliferative reaction against the lens of the semi-xenogeneic frog, they did not exhibit enhanced proliferation against the syngeneic lens at all. On the other hand, splenocytes from naive adult frogs that had been enucleated at stage 43 proliferated intensely in response to the syngeneic lens. Similar significantly high proliferation against the syngeneic lens was also observed in splenocytes from naive frogs enucleated at stages 56 and 66 (Fig. 3).

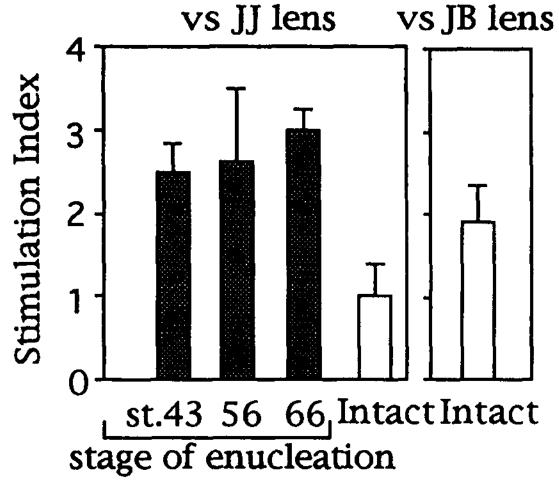

FIGURE 3 Responses of splenocytes from frogs enucleated during larval stages, against syngeneic or semi-xenogeneic lenses. JJ splenocytes were co-cultured with the syngeneic JJ lens or the semi-xenogeneic JB lens as described in Materials and Methods. Splenocytes were obtained from naive adult frogs that had been enucleated at stages 43, 56, 66, or from intact frogs. The number of BrdU-incorporated proliferating splenocytes is shown as the relative proliferating rate (Stimulation Index) on the 5th day. The actual proliferating cells in the control culture was 3.51%, where the stimulation index was 1. Values represent means ± standard deviations (SD) obtained from three replicate cultures

Moreover, splenocytes from frogs enucleated even after metamorphosis gradually began to proliferate vigorously against the syngeneic lens, as a function of the period after eye removal (Fig. 4). These frogs having proliferative splenocytes accepted the grafted lens, like those enucleated at stage 56 or later (Table I). Interestingly, even after acceptance of the grafted lens, their splenocytes remained highly proliferative against the syngeneic lens *in vitro* (Fig. 4). A similar proliferative response was not observed in splenocytes from frogs that had been enucleated at stage 43 and thymectomized at stages 45 to 47 or frogs that had only one eye removed at stage 43 (Fig. 5). To investigate whether antigen-presenting cells (APCs) were required for the proliferative response, we eliminated APCs from splenocytes using nylon wool columns. The splenic leukocytes without APCs did not proliferate against the lenses in co-culture (Fig. 5).

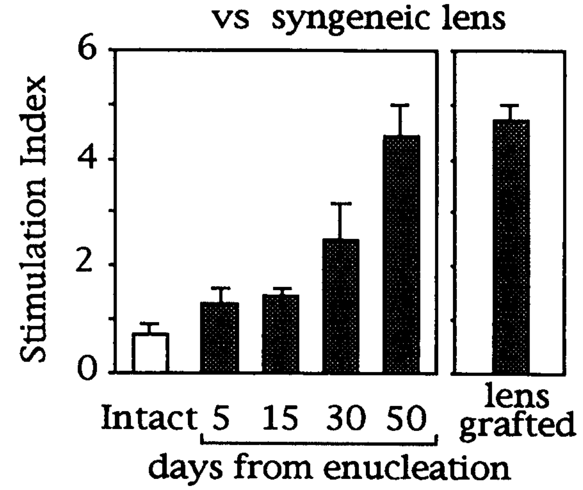

FIGURE 4 Responses of splenocytes from frogs enucleated after metamorphosis. Splenocytes were prepared from intact adult frogs or from enucleated frogs on the 5th, 15th, 30th, and 50th day after both eyes had been removed. Splenocytes from adult frogs grafted with a syngeneic lens on the 30th day after eye removal were also cultured with another syngeneic lens on the 50th day after lens grafting. The number of BrdU-incorporated proliferating splenocytes is shown as the relative proliferating rate (Stimulation Index) on the 5th day. The actual proliferating cells in the control culture was 3.21%, where the stimulation index was 1. Values represent mean ± SD obtained from three replicate cultures

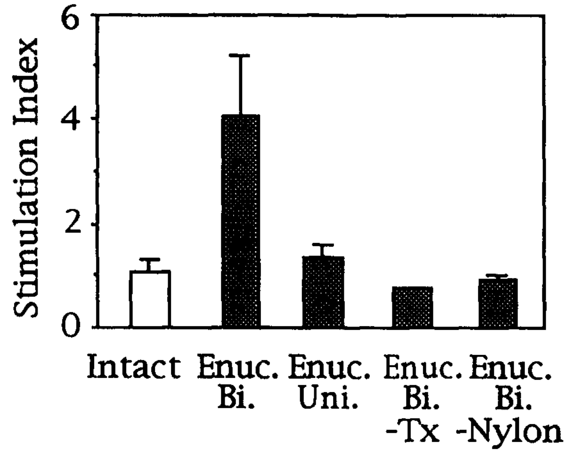

FIGURE 5 Effect of unilateral enucleation, thymectomy, and removal of APCs on proliferative responses. Splenocytes were prepared from intact adult frogs (Intact), from those bilaterally enucleated at stage 43 (Enuc.Bi.), unilaterally enucleated at stage 43 (Enuc.Uni.), or from those bilaterally enucleated at stage 43 and thymectomized at stages 45 to 47 (Enuc.Bi.-Tx). Some splenocytes obtained from frogs bilaterally enucleated at stage 43 were passed through nylon wool columns (Enuc.Bi.-Nylon) as described in Materials and Methods. The number of BrdU-incorporated proliferating splenocytes is shown as the relative proliferating rate (Stimulation Index) on the 5th day of culture. The actual proliferating cells in the control culture was 3.60%, where the stimulation index was 1. Values represent means ± SD obtained from three replicate cultures, except for those of splenocytes from frogs enucleated at stage 43 and thymectomized at stages 45 to 47 which were obtained from two replicate cultures

To investigate what antigens are recognized by splenocytes, a lens with exuded lens fiber, an isolated lens epithelium (with the lens capsule), and a piece of exuded lens fiber without the epithelium were prepared manually using fine forceps. The obtained lens tissues were co-cultured with splenocytes from intact frogs or from those enucleated at stage 43. No proliferative response was observed in splenocytes from intact frogs against any lens tissues. Splenocytes from frogs enucleated at stage 43 proliferated significantly in response to the lens with epithelium but did not proliferate in response to the lenses without lens epithelium (Fig. 6). Exuded lens fiber was shown to have no inhibitory effects on splenocyte proliferation (Fig. 6).

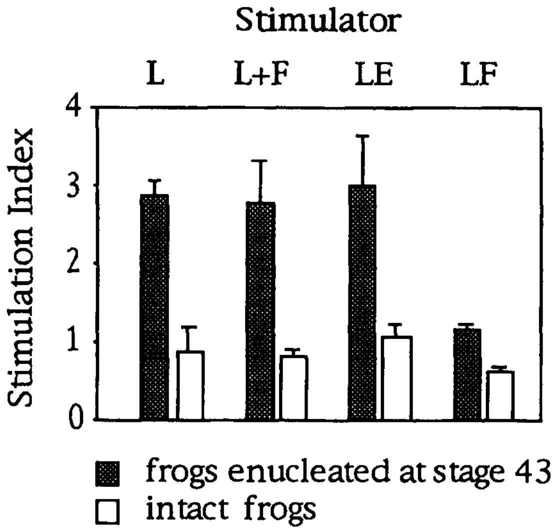

FIGURE 6 Proliferation of splenocytes in response to antigens on the lens epithelial cells. Splenocytes were co-cultured with syngeneic intact lens (L), disrupted lens with discharged lens fiber attached to the surface (L+F), isolated lens epithelium (plus lens capsule) (LE), or lens fiber (LF) as described in Materials and Methods. Splenocytes were prepared from intact adult frogs and from those enucleated at stage 43. The number of BrdU-incorporated splenocytes is shown as the relative proliferating rate (Stimulation Index) on the $5^{th}$ day. The actual proliferating cells in the control culture was 4.00%, where the stimulation index was 1. Values represent means ± SD obtained from two replicate cultures

### DISCUSSION

The present experiments were designed to find possible evidence of a potentially destructive immune response against "self" lenses in frogs enucleated at an early larval stage. It had long been thought that the antigens in the eye remained "hidden" from the immune system throughout life. However, recent mammalian investigations have demonstrated that the antigens in the eye are accessible to the immune system (Griffith et al., 1995; Streilein, 1996). Thus, it is plausible to hypothesize that the immune system of frogs interacted with the lens antigens until enucleation. We have investigated the response of adult frogs that had been enucleated during ontogeny. The lens epithelial cells begin to be formed at stages 33 to 34 and flatten at stage 45 in *Xenopus* (Nieuwkoop and Faber, 1956). Based on this information about the development of the immune system and the eye, we enucleated the animals at four stages: (1) at stage 43, before the occurrence of lymphoid differentiation in the thymus; (2) at stage 56, when the immune system of the tadpole-type is established; (3) at stage 66, just after they metamorphose; and (4) at more than 2 months after they metamorphose, when the adult-type immune system matures (cf. Du Pasquier, 1982 ; Flajnik et al., 1987).

In the lens transplantation experiments, only frogs that had been enucleated at stage 43 exhibited intense responses against the grafted syngeneic lenses accompanied by inflammation (Fig. 1; Table I). The lens epithelial cells were destroyed and eventually disappeared (Fig. 2). A similar destruction or disappearance was not observed in any intact frogs or in frogs with only one eye removed. In addition, no damage was observed in the grafted lenses even in frogs enucleated at stages 56, 66, or after metamorphosis (Table I). Those enucleated at stage 48 displayed essentially the same results as those operated later (data not shown). We have concluded that this destruction and disappearance of lens epithelial cells is a result of the immune response. One of the reasons for this conclusion is that these responses were accompanied by heavy infiltration by lymphocytes and phagocytes (Fig. 1; cf. Hadji-Azimi et al., 1987). More directly, the rejection was shown to be thymus-dependent, because the response was not observed in frogs that had been enucleated at stage 43 and thymectomized at stages 45 to 47 (Table I). It may be due to an incomplete thymectomy that the grafted lens was destroyed in one exceptional case out of nine. Although the histological examination did not give us a convincing evidence for secondary rejection response to lens graft, enhanced *in vitro* proliferative response was observed after the completion of rejection in animals enucleated at stage 43 and lens grafted in adult life (see what follows). Our results, that is, the grafted lenses were rejected thymus-dependently only by frogs enucleated before the occurrence of lymphoid differentiation in the thymus, agree with those by Triplett (1962) and thus support his theory that self antigens removed prior to development of the immune system cannot be regarded as "self."

In order to quantitate the responses of enucleated frogs against syngeneic lenses, we tried to co-culture a lens and splenocytes. The splenocytes from intact frogs or from those with only one eye removed did not respond significantly against the syngeneic lenses (Fig. 3). However, splenocytes from frogs enucleated at any larval stage (stages 43, 56, or 66) proliferated intensely in response to the syngeneic lenses (Fig. 3). Although the positive reaction was observed against semi-xenogeneic lens in intact frogs, we have no data showing that this reaction was evoked by MHC antigens. Interestingly, splenocytes from frogs enucleated even after metamorphosis proliferated vigorously against the syngeneic lenses on the 30$^{th}$ or 50$^{th}$ day after enucleation (Fig. 4). There was no suppressive effect of lens grafting on the response of splenocytes in these frogs. Even after acceptance of the grafted lenses (cf. Table I), the splenocytes remained highly proliferative against the syngeneic lenses (Fig. 4). The surprising fact may be that the *in vitro* proliferative responses was observed in the absence of priming with lens graft. However, the spleen cells obtained from lens-rejected frogs enucleated at stage 43 showed heightened proliferation at 50 days after the lens graft, than before the lens grafting (data not shown). Like rejection of grafted lens, proliferation of splenocytes against the lenses was not observed in frogs that were enucleated at stage 43 and thymectomized at stages 45 to 47, indicating that this proliferative response was also thymus-dependent (Fig. 5).

The question that we must consider is what antigens in the lens induce the observed immune responses and how they induce these responses. Although the lens epithelium in the grafted lenses was rejected by frogs enucleated at stage 43, lens fiber within the lenses remained apparently intact beyond the 100$^{th}$ day after grafting (data not shown). Also in co-culture, splenocytes from frogs enucleated at stage 43 proliferated against only the lenses with epithelium (Fig. 6). It is well known that crystallin, contained in lens fiber, is expressed also in non ocular tissues in *Xenopus* (Smolich et al., 1994). If the immune system of an enucleated frog can interact with crystallin that is expressed in non ocular tissues, it is plausible that the lens fiber is recognized as "self." Therefore, we believe that the unknown antigens recognized as "non self" exist on the lens epithelium.

Since the proliferative responses are thymus-dependent in co-culture, it can be assumed that main cell populations that proliferate in response to the lenses are T lymphocytes (Fig. 5; cf. Du Pasquier and Horton, 1976). Based on the difference in the results of transplantation and co-culture experiments, we assume that the proliferative T lymphocytes and the cytotoxic cells are different cell populations. The candidates for cytotoxicity are either cytotoxic T lymphocytes, B lymphocytes, NK cells, granulocytes, or macrophages. Since the rejections were also thymus-dependent and accompanied by infiltration of T lymphocytes (including CD8-positive cells), it is clear that T lymphocytes play an important role in rejection. It is reasonable to assume that effector cells are either in one of the cytotoxic T lymphocytes, the T-dependent NK cells, or the B lymphocytes activated by T lymphocytes, or possibly all.

It is well known that T lymphocytes are restricted to MHC molecules. Since APCs are required for the proliferative responses in the co-culture, it is likely that APCs phagocytose lens epithelial cells, indirectly present lens antigens on their MHC class II molecules to T lymphocytes, and activate T lymphocytes. Based on our immunohistological observations (data not shown), neither MHC class I nor class II molecules were expressed in the lenses of the frogs, as was the case in mice (Shaughnessy and Wistow, 1992). On the other hand, induction of class I and class II MHC molecule by immune cells is not a rare phenomenon in animal species (Kirby et al., 1993; Lin et al., 1993; Egwuagu et al., 1994). Likewise in the present study, both MHC class I and class II molecules seemed weakly expressed in some lens epithelial cells that were surrounded by many leukocytes, after grafting or co-culture (data not shown). It is apparent, however, that the induced MHC molecules on the lens epithelial cells, if any, were not sufficient to directly activate proliferative T lymphocytes without APC addition (Fig. 5).

The fact that the grafts of whole eyeballs were never rejected even in frogs enucleated in early larval life is in accord with the results of a previous report (Rollins-Smith et al., 1990). It is well known that the eyeball is an immune privilege tissue where grafts of even foreign tissues can expand their survival in mammals (Griffith et al., 1995). Although there is no report of *Xenopus* having an immune privilege region, the overall difference in the results of lens and whole eyeball transplantation supports the idea that the eyeball is the immune privilege tissue in this animal, too.

It was also reported that tadpoles or frogs in which the hypophysis and the thyroids were removed as embryos could not reject syngeneic implants of the removed organs (Rollins-Smith and Cohen, 1982a, 1982b, 1983; Maeno and Katagiri, 1984). However, it was suggested that the tadpoles and the frogs with their hypophysis or with thyroids removed do not appear to be suitable as recipients (Flajnik et al., 1987; Rollins-Smith et al., 1988; Rollins-Smith and Blair, 1993), and the organs have too weak transplantation antigens to be rejected in *Xenopus* (Maeno and Katagiri, 1984). These results imply in turn that lenses having unique antigens specific to the lens epithelium are more suitable than hypophysis or thyroids as test organs.

In conclusion, the present results clearly support the hypothesis that the immune system can learn not to respond to antigens on lens epithelial cells during early larval life. Intact frogs recognized the syngeneic lenses completely as "self," and never showed positive responses against the lenses both *in vivo* (cytotoxicity) and *in vitro* (proliferation of splenocytes). However, frogs that had been enucleated in early larval life came to recognize the syngeneic lenses as "non self", and showed cytotoxic and proliferative responses against the syngeneic lenses. Interestingly, sequential enucleation experiments indicated that frogs enucleated in late larval life, or even after maturation of the immune system, showed a proliferative response against lenses but accepted the grafted lenses. These frogs seemed to change to recognize the syngeneic lenses partially as "non self," resulting in a situation of split-tolerance (cf. Sakuraoka and Tochinai, 1993). We do not think that this means that tolerance of the proliferating T cells develops very late in larval life, but rather the maintenance of the tolerant state is dependent on the presence of a whole eyeball or lens throughout life. Thus, once the tolerogenic eyes are removed, the tolerant state will be easily and promptly broken down resulting in the previously mentioned split-tolerance. In this connection, it is interesting to note the observation reported previously that even intact frogs as well as embryonically enucleated frogs responded equally well in anti-self-lens proliferative and antibody responses (Rollins-Smith et al., 1990). Taken together, we can conclude that tolerance to self lens in this species is very unstable rather than non existent.

### MATERIALS AND METHODS

### Experimental Animals

The animals used in the present study were from the MHC identical J strain (haplotype; JJ) of *Xenopus laevis,* MHC identical partially inbred colony *Xenopus borealis* (BB), and their hybrids (JB). Fertilized eggs of J strain were obtained by hormone-induced matings, and the hybrid was made by artificial insemination between *X. laevis* eggs and *X. borealis* sperm (Sakuraoka and Tochinai, 1993). Larvae and adults were reared at 23°C. Tadpoles were staged according to the Normal Table of Nieuwkoop and Faber (1956). It has been repeatedly demonstrated in our laboratory that all members of J strain animals mutually accept any tissues and organs including skin, eye, and lens.

### Enucleation (Eye Removal)

From animals that had developed to stages 43, 56, and 66 and frogs older than 2 months after metamorphosis, whole eyeballs were extirpated with scissors or a scalpel under a stereomicroscope.

### Thymectomy

Thymectomy was performed on larvae at 4 to 5 days old (stages 45 to 47) with a electrolytically sharpened, very fine needle attached to an electoromicrocautery apparatus (Horton and Manning, 1972). Absence of the thymus was confirmed by external observation of larvae at stages 50 to 55.

### Eye or Lens Transplantation

Enucleated frogs were transplanted with syngeneic eyes from similarly aged or slightly younger adult members of the same strain, according to the method of Rollins-Smith et al. (1990). Briefly, intact whole eyeballs were dissected out and transplanted under the dorsal skin. For external observation under a stereomicroscope, a small area of the skin above the grafted eye was cut off. The transplanted eyes were fixed with Bouin's solution, embedded in paraffin, serially sectioned, and stained with hematoxylin and eosin for histological examination.

For transplantation, carefully removed lenses freed from the eyeballs were grafted through a small mid line slit made in the dorsal skin to a space made between the muscle membrane and the muscle *(longissimus dorsi).* The transplanted lenses were observed histologically in the same way as the eyes.

### Immunohistochemistry

Expression of MHC class I and class II in the lenses was examined immunohistochemically using monoclonal antibodies (mAbs) TB17 (IgG1, anti-*Xenopus* MHC class I) and mAbs AM20 (IgG, anti-*Xenopus* MHC class II) (Flajnik et al., 1990; Harding et al., 1993) on cryostat sections. The inflammatory cells that had accumulated around the grafted lenses were characterized immunohistochemically using mAbs XT-1 (IgG2 with specificity for a 120kDa *Xenopus* T-cell antigen) and mAbs AM22 (IgM, anti-*Xenopus* CD8) (Nagata, 1988; Flajnik et al. 1990). The mAbs TB17, AM20, and AM22 were generously provided by Dr. Martin Flajnik, and mAbs XT-1 was provided by Dr. Saburo Nagata. The second antibodies used were Cy3-conjugated goat anti-mouse IgG, M polyclonal antibodies (Chemicon International, Temicula, CA). After further staining with quinacrine dihydrocholoride, the sucrose-mounted preparation was observed under an epifluorescence microscope.

### Co-Culture of a Lens and Splenocytes

A lens and splenocytes were co-cultured according to the method for co-culturing skin and splenocytes with some modifications ( Izutsu et al., 1996). Intact lenses from frogs aged about 1 or 2 months were used. Each lens or lens tissue attached to a small piece of 0.22-micrometer filter (Millipore) was placed in a 100-microlitre culture medium [70% L-15 (Gibco, Grand Island, NY) supplemented with 10 mM $NaHCO_3$, 10 mM HEPES, 100 units/ml penicillin G, 100 microg/ml streptomycin sulfate, and 10% heat-inactivated FCS (Filtron, Pty., Australia)].

Five $\times$ $10^5$ splenic leukocytes prepared in 70% L-15 and a lens were co-cultured in a 96-well U-bottom culture dish containing 200-microliter culture medium at 28°C in a humidified atmosphere of 5% $CO_2$ and 95% air. After 4 days of culture, the proliferation of leukocytes was assessed by incubating the cultures with 500 mM 5-bromo-2′-deoxyuridine (BrdU) (Sigma Chemical, St. Louis) for an additional 24 hours according to the previous experiments (Sakuraoka and Tochinai, 1993; Izutsu et al., 1996). The incubation period was determined in order to give a reliable count by microscopic counting. Splenocytes harvested from each culture were fixed in absolute methanol and were smeared on slide glasses. The smears were serially treated with anti-BrdU mouse monoclonal antibody (Sanbio, Netherlands) and Rhodamine-conjugated goat anti-mouse IgA+IgG+IgM antibody (Cappel, England). Nuclei of the preparations were stained with 0.025% quinacrine dihydrochloride and observed under an epifluorescence microscope (Nikon EFD2; Nikon, Tokyo). Cells that had incorporated BrdU into their nuclei were counted as proliferating cells. The relative rate in co-culture (Stimulation Index) was calculated on more than 1000 cells, as the mean proliferating rate of splenocytes of the same origin cultured without a lens was assumed to be 1. The actual numbers of the proliferating cells in control cultures were usually 2 to 6% of the total (20 to 60 positively stained cells in

1000), depending on the experiments. In some experiments, splenic cell suspensions were passed through nylon wool columns (Wako Pure Chemical Industries, Japan) to eliminate antigen-presenting cells (Julius et al., 1973).

### *References*

Du Pasquier L. (1982). Ontogeny of immunological functions in amphibians. In: The Reticuloendothelial System: A Comprehensive Treatise 3. Phylogeny and Ontegeny. (Vol. 3) Cohen N., and Sigel M.M., Eds. New York: Plenum Press. pp. 633–657.

Du Pasquier L., and Horton J.D (1976). The effect of thymectomy on the mixed leukocyte reaction and phytohemagglutinin responsiveness in the clawed toad *Xenopus laevis*. Immunogenetics **3**: 105–112.

Egwuagu C.E., Sztein J. Chan Chi-Chao, Reid W., Mahdi R., Nussenblatt R.B., and Chepelinsky A.B. (1994). Ectopic expression of gamma interferon in the eyes of transgenic mice induces ocular pathology and MHC class II gene expression. Invest. Ophthalmol. Vis. Sci. **35**(2): 332–341.

Flajnik M.F., Ferrone S., Cohen N., and Du Pasquier L. (1990). Evolution of the MHC: Antigenicity and unusual tissue distribution of *Xenopus* (Frog) class II molecules. Mol. Immunol. **27**(5): 451–462.

Flajnik M.F., Hsu E., Kaufman J.F., and Du Pasquier L. (1987). Changes in the immune system during metamorphosis of *Xenopus*. Immunol. Today **8**(2): 58–64.

Griffith T.S., Brunner T., Fletcher S.M., Green D.R., and Ferguson T.A. (1995). Fas ligand-induced apoptosis as a mechanism of immune privilege. Science **270**: 1189–1192.

Hadji-Azimi I., Coosemans V., Canicatti C., and Perrenot N. (1987). Atlas of adult *Xenopus laevis laevis* hematology. Dev. Comp. Immunol. **11**: 807–874.

Harding F.A., Flajnik M.F., and Cohen N. (1993). MHC restriction of T-cell proliferative responses in *Xenopus*. Dev. Comp. Immunol. **17**: 425–437.

Horton J.D., and Manning J.M. (1972). Response to skin allografts in *Xenopus laevis* following thymectomy at early stages of lymphoid organ maturation. Transplantation **14**(2): 141–154.

Izutsu Y., Yoshizato K., and Tochinai S. (1996). Adult-type splenocytes of *Xenopus* induce apoptosis of histocompatible larval tail cells *in vitro*. Differentiation **60**: 277–286.

Julius M.H., Simpson E., and Herzenberg L.A. (1973). A rapid method for the isolation of functional thymus-derived murine lymphocytes. Eur. J. Immumol. **3**: 645–649.

Kirby J.A., Rajasekar M.R., Lin Y., Proud G., and Taylor R.M.R. (1993). Interaction between T lymphocytes and kidney epithelial cells during renal allograft rejection. Kidney International **43** (Suppl. 39): S124-S128.

Lin Y., Proud G., Taylor R.M.R., and Kirby J.A. (1993). Renal allograft rejection: protection of renal epithelium from natural killer cells by cytokine-induced up-regulation of class I major histocompatibility antigens. Immunol. **79**: 290–297.

Maeno M., and Katagiri Ch. (1984). Elicitation of weak immune response in larval and adult *Xenopus laevis* by allografted pituitary. Transplantation **38**(3): 251–255.

Nagata S. (1988). T cell-specific antigen in *Xenopus* identified with a mouse monoclonal antibody: biochemical characterization and species distribution. Zool. Sci. **5**: 77–83.

Nieuwkoop P.D., and Faber J. (1956). Normal table *of Xenopus laevis* (Daudin) Amsterdam: North-Holland.

Rollins-Smith L.A., and Blair P.J. (1993). The effects of corticosteroid hormones and thyroid hormones on lymphocyte viability and proliferation during development and metamorphosis of *Xenopus laevis*. Differentiation **54**:155–160.

Rollins-Smith L.A., and Cohen N. (1982a) Self-pituitary grafts are not rejected by frogs deprived of their pituitary anlagen as embryos. Nature **299**: 820–821.

Rollins-Smith L.A., and Cohen N. (1982b). Neither self-eye nor self pituitary implants are rejected by frogs deprived of their eye or pituitary anlagen as embryos. Ann. New York Acad. Sci. **392**: 413–414.

Rollins-Smith L.A., and Cohen N. (1983). The Triplett phenomenon revisited: self-tolerance is not confined to the early developmental period. Transplantation Proc. **XV**(1): 871–874.

Rollins-Smith L.A., Parsons S.C.V. and Cohen N. (1988). Effects of thyroxine-driven precocious metamorphosis on maturation of adult-type allograft rejection responses in early thyroidectomized frogs. Differentiation **37**(3): 180–185.

Rollins-Smith L.A., Parsons S.C.V., and Cohen N. (1990). Immune responses of intact and embryonically enucleated frogs to self-lens antigens. J. Immunol. **145**(10): 3262–3267.

Sakuraoka J., and Tochinai S. (1993). Demonstration of cells involved in rejection of tolerogenic grafts in tolerant *Xenopus*. Dev. Comp. Immunol. **17**: 439–447.

Shaughnessy M., and Wistow G. (1992). Absence of MHC gene expression in lens and cloning of dbpB/YB-1, a DNA-binding protein expressed in mouse lens. Curr. Eye Res. **11**(2): 175–181.

Smolich B.D., Tarkington S.K., Saha M.S., and Grainger R.M. (1994). *Xenopus* g-crystallin gene expression: Evidence that the g-crystallin gene family is transcribed in lens and nonlens tissues. Mol. Cell. Biol. **14**(2): 1355–1363.

Streilein J.W. (1996). Peripheral tolerance induction: lessons from immune privileged sites and tissues. Transplantation Proc. **28**(4): 2066–2070.

Triplett E.L. (1962). On the mechanism of immunologic self recognition. J. Immunol. **89**: 505–510.

